# Factors associated with HIV infection among children born to mothers on the prevention of mother to child transmission programme at Chitungwiza Hospital, Zimbabwe, 2008

**DOI:** 10.1186/1471-2458-13-1181

**Published:** 2013-12-14

**Authors:** Stella Ngwende, Notion T Gombe, Stanley Midzi, Mufuta Tshimanga, Gerald Shambira, Addmore Chadambuka

**Affiliations:** 1 Department of Community Medicine, University of Zimbabwe, Harare, Zimbabwe; 2 Ministry of Health and Child Care, Harare, Zimbabwe

**Keywords:** HIV infection, Risk factors, PMTCT, Chitungwiza Hospital

## Abstract

**Background:**

Zimbabwe is one of the five countries worst affected by the HIV/AIDS pandemic with HIV infection contributing increasingly to childhood morbidity and mortality. Among the children born to HIV positive mothers participating in the PMTCT programme, 25% tested positive to HIV. We investigated factors associated with HIV infection among children born to mothers on the PMTCT programme.

**Methods:**

A 1:1 unmatched case–control study was conducted at Chitungwiza Hospital, Zimbabwe, 2008. A case was defined as a child who tested HIV positive, born to a mother who had been on PMTCT programme. A control was a HIV negative child born to a mother who had been on PMTCT programme. An interviewer-administered questionnaire was used to collect data on demographic characteristics, risk factors associated with HIV infection and immunization status.

**Results:**

A total of 120 mothers were interviewed. Independent risk factors associated with HIV infection among children included maternal CD4 count of less than 200 during pregnancy [aOR = 7.1, 95% CI (2.6-17)], mixed feeding [aOR = 29, 95% CI (4.2-208)], being hospitalized since birth [aOR = 2.9, 95% CI (1.2-4.8)] whilst being exclusively breast fed for less than 6 months [aOR = 0.1 (95% CI 0.03-0.4)] was protective.

**Conclusions:**

HIV infection among children increased if the mother’s CD4 count was ≤200 cells/μL and if the child was exposed to mixed feeding. Breastfeeding exclusively for less than six months was protective. We recommended exclusive breast feeding period for the first six months and stop breast feeding after 6 months if affordable, sustainable and safe.

## Background

Worldwide, Acquired Immunodeficiency Syndrome (AIDS) accounts for three per cent of deaths in children under five years of age, and six per cent of those in sub-Saharan Africa, where AIDS has become one of the major killers of young children. At least 1600 infants are infected with Human Immunodeficiency Virus (HIV) everyday and more than 600,000 infants are infected by the virus annually. About 90% of these infections occur in developing countries, mainly Sub-Saharan Africa [1].

It has been estimated that globally 34 million people were living with HIV at the end of 2011. There was 2.5 million new HIV infections and this included 330 000 among children less than 15 years. Most of these children were from sub-Saharan Africa, and mother-to-child transmission during pregnancy, on delivery and during breastfeeding were the major route of infection. It is believed that about two thirds are infected during pregnancy and around the time of delivery, and about one third are infected through breastfeeding [2]. Interventions have reduced MTCT of HIV to less than 2% in high income countries [3,4] but this rate remains high in resource poor countries ranging between 20% and 45% [5].

Zimbabwe is one of the five countries worst affected by the HIV/AIDS pandemic in the world. According to Zimbabwe Demographic Health Survey (ZDHS) 2010/11, the national adult HIV prevalence is 15% with 17% in the urban and 15% in the rural areas. The prevalence is highest among women aged 30–39 and men aged 45–49 and is higher among females (18%) than in males (12%) [6]. In 2011, Zimbabwe adopted the WHO PMTCT guidelines The Ministry of Health in Zimbabwe established the Prevention of Mother to Child Transmission (PMTCT) Programme as part of continuing strategies for dealing with the epidemic and interventions to reduce mother to child transmission. Zimbabwe has committed itself to the goal of eliminating new paediatric HIV and keeping their mothers and families alive, in line with the Global Plan which was launched in June 2011. The goal of the strategy is the elimination of new HIV infections among children by 2015. The national goals of Zimbabwe PMTCT programme to attain MDGs are 1) reduce MTCT to less than 5% by 2015, 2) Improving the survival of mothers and children in the context of HIV [7]. The objectives of the PMTCT programme are to make available HIV voluntary and confidential counselling and testing (VCCT) to all pregnant women so that they may make fully informed decisions; and to administer a short-course drug regimen for HIV positive pregnant women to reduce mother to child HIV transmission [8].

Zimbabwe is on track to meet the ambitious 2015 target of elimination of new HIV infections in children and keeping the mothers alive [9]. The goal of any programme on prevention or reduction of HIV infected children is to prevent HIV infection in parents-to-be and to prevent unplanned pregnancies in HIV-infected women. Among pregnant women already infected with HIV, antiretroviral prevention therapy, safe delivery practices and safe infant- feeding options to reduce the risk of mother-to-child transmission of HIV should be provided.

The risk of transmission by an infected mother occurring before or during birth (without interventions to reduce transmission) is 15–25%. In Zimbabwe like any other country in sub-Saharan Africa, breastfeeding is the norm [10] with 97% of children reported as ever breastfed [6]. In view of the fact that a third of HIV infections occur during breastfeeding, this presents a challenge to HIV control programs on what options to recommend to women. HIV positive mothers have to decide whether to breastfeed, which carries a risk of transmitting HIV through breast milk, or to use formula which reduces the risk of HIV transmission but usually beyond reach of most women and is associated with higher rate of infant mortality from diarrhoeal disease [11]. Current WHO recommendation is that HIV positive women breastfeed exclusively for the first six months, and introduce other foods thereafter, unless replacement feeding with formula meets the criteria of being acceptable, feasible, affordable sustainable and safe [12,13]. In Zimbabwe, only 1 in 3 children under six months are being exclusively breastfed [6].

Studies have shown that exclusive breastfeeding i.e. giving the infant breast milk only and no other liquids or solids for the first six months of life, reduces the rate of MTCT to less than one fourth compared to mixed feeding [14-16] and HIV free survival of the infants [17]. Exclusive breastfeeding by mothers is a challenge to adhere to during the first six months for most women and therefore predisposed the infant to the risk of HIV infection especially for mothers that have responsibilities that take them away from their children during the day. Caregivers may introduce other foods when children are left under their care thereby increasing risk of HIV transmission [18]. The transmission risk can be reduced by antiretroviral prophylaxis [19-22] and by avoidance of breastfeeding if it is acceptable, feasible, affordable sustainable and safe (AFASS). The overall rate of transmission can go up to 20% if an HIV infected mother breastfeeds for 18–24 months [23]. Maternal factors are also associated with increased risk of transmission during breastfeeding. Recent maternal infection with HIV raises the risk of transmission through breastfeeding to twice that of a woman with earlier established infection, owing probably to high viral load associated with recent infection [24].

In this study, we determined factors associated with HIV transmission, determined the infant feeding choices of the HIV infected mothers on PMTCT.

## Methods

### Design

A 1:1 unmatched case–control study was conducted at Chitungwiza Central Hospital from June to August 2007. Frequency matching of the cases and controls on age to take care of the possible differences in exposure that would arise by difference in age and still be able to analyse the age variable. A case was defined as a child who tested HIV positive before or at age 18 months and born to a mother who had been on the PMTCT program. A control was any child born to a mother who had been on the PMTCT program and was HIV negative at 18 months.

### Setting and context

Chitungwiza Central Hospital offers to HIV-positive mothers access a package of services that promotes safe delivery and ensures safe postnatal care and support for both the baby and the mother, which included voluntary HIV counselling and testing to pregnant women and a short course drug regimen for all HIV positive women to prevent mother to child transmission. The drug of choice was nevirapine given as a single dose to pregnant mothers during delivery at onset of labour according to the national guidelines. The women were given nevirapine to take home and swallow at onset of labour. The drug was also administered as a single dose to the baby soon after delivery and up to 72 hours post-delivery.

Among the children born to HIV positive mothers participating in the PMTCT programme, 25% tested positive to HIV. We investigated factors associated with HIV infection among children born to HIV positive mothers already on the PMTCT programme at Chitungwiza Hospital. Study Population: Our study population was mothers who were on PMTCT programme whose babies were 18 months or more and had been tested for HIV at Chitungwiza Central Hospital. A mother was on the PMTCT programme if she had received HIV testing and counselling, had a positive result and received both maternal and infant prophylaxis. All mothers on the PMTCT programme and who visited the Chitungwiza Hospital Opportunistic Infections (OI) clinic for routine visits during the study period were eligible for enrolment into the study.

### Inclusion and exclusion criteria

A mother who was on the PMTCT programme with a child who had been tested for HIV was included in the study. A mother who was not on PMTCT programme or a mother with a child 18 months and above who had not tested for HIV was excluded together with all those who declined to participate.

### Selection of study participants

Purposive sampling of mothers, with children, who had been offered PMTCT, was done. A line list of mothers who had tested HIV positive and delivered at Chitungwiza Central Hospital and were on the PMTCT programme was developed from the antenatal care, delivery and PMTCT registers to enable identification of potential mothers. If the mothers satisfied the inclusion criteria, they were asked if they were willing to participate in the study upon presenting at the Opportunistic Infection Clinic. We did not test the child for HIV but relied on records in the hospital registers for their HIV status. Women who visited the hospital during the study period fitting our inclusion criteria and consenting to participate were enrolled in the study.

### Sample size

We used the StatCalc function of EPI Info™ statistical software, and calculated at 95% confidence interval, 80% power. We made the assumption that exposure to HIV in the children who were HIV negative was 25% and OR to be 3.16 [25]. This resulted in the minimum sample size being 60 cases and 60 controls.

### Data collection

Data was collected from the mothers at the hospital by the researchers using a pretested structured interviewer administered questionnaire. A structured questionnaire was administered to the mother in the local language (Shona) to obtain information on the infant feeding choices, nevirapine non- adherence at delivery for both mother and baby, and common childhood morbidities suffered by the baby during childhood. Information on mode of delivery and duration of labour, maternal factors like CD4 count and immunization status of the children were obtained from the maternal labour records and child health cards using a data abstraction form that was designed for this purpose. Counselling services were provided by Primary Care Counsellors employed at the Hospital. We also reviewed pharmacy records to check on the availability of drugs during the period we were studying.

Pretesting of the questionnaire was done at St Mary’s Clinic Chitungwiza and adjustments were made where necessary.

### Data analysis

We used EPI Info™ statistical software to analyse data. We generated frequencies, proportions and means of variables. We calculated odds ratios and their 95% confidence intervals. Stratified analysis was conducted to identify potential confounding variables and assess for interaction. We performed stepwise forward logistic regression to determine independent factors associated with HIV infection. All factors that had a p-value ≤0.25 on bivariate analysis were fitted into the logistic regression model. We assumed normality, a linear relationship between variables and equal variances within each group.

### Definitions

Exclusive breastfeeding in this study was defined as giving the infant only the mother’s milk for the first six months. Prescribed medicines/vitamin supplements/oral vaccines were allowed in the definition of exclusive breastfeeding. Mixed feeding is giving the baby under the age of six months other liquids and / or solid food besides breast milk.

#### Ethical considerations

Permission to carry out the study was obtained from Chitungwiza Hospital Management and the Health Studies Office (HSO). Ethical approval was granted by the Medical Research Council of Zimbabwe (MRCZ) as well as the Chitungwiza Hospital Institutional Review Board. Informed written consent was obtained from all study participants. Confidentiality was assured and maintained throughout the study. Names of participants were not written down and participation was voluntary as no payment or cohesion was used. Information was treated as confidential and results of the study were disseminated to Chitungwiza Hospital Management and the Health Studies Office.

## Results

### Demographic characteristics of mothers on PMTCT programme and their children

A total of 120 mothers were interviewed. Sixty were cases and 60 were controls. Thirty seven (62%) cases and 44 (73%) controls were married. Forty four cases (73%) and 48 (80%) controls had attained secondary education. The median age for the mothers was 28 years (Interquartile range (IQR) 24–31) for cases and 29 years (IQR 25–33) for controls. The median age for the children was 17 months (IQR 12–27) for cases and 24 months (IQR 18–35)) for controls. Most women had their children tested for HIV between six weeks to three months. The demographic characteristics of the respondents are summarized in Table 1.

**Table 1:** Demographic characteristics of respondents

	**Cases**	**Controls**
	**n(%) = 60**	**n(%) =60**
**Marital status**		
Married	37 (62)	44 (73)
Divorced	11 (18)	4 (7)
Single	6 (10)	8 (13)
Widowed	6 (10)	4 (7)
**Level of education**		
Primary school	16 (27)	12 (20)
Secondary school	44 (73)	48 (80)
**Religion**		
Apostolic	8 (13)	8 (13)
Catholic	26 (43)	13 (22)
Protestant	12 (20)	19 (32)
Pentecostal	14 (23)	18 (30)
None		2 (3)
**Median age of mothers (years)**	28 (IQR 24–31)	29 (IQR 25–33)
**Median age of children (months)**	17 (IQR 12–27)	24 (IQR 18–35)

### Maternal factors

The median CD4 count of the cases was 180 cells/μL (IQR 131–361) while that of the controls was 357 cells/μL (IQR 229–435). Thirty one (52%) of the cases had a CD4 count of less or equal to 200 cells/μL compared to 10 (17%) of the controls. Eighteen (30%) of the cases and 10 (17%) of the controls had contracted a sexually transmitted infection (STI) during the last pregnancy. The most common STI was the Vaginal Discharge Syndrome (VDS) with 9/18 (50%) cases and 7/10 (70%) controls contracting it. The most common form of delivery was the vaginal delivery, 52 (87%) cases and 50 (83%) controls chose it and all the deliveries were conducted by skilled midwives. Sixty three of the respondents were given a choice on the mode of delivery. Thirty six (58%) of these mentioned that it was cheap option whilst 18 (29%) preferred the normal delivery as they were afraid that caesarean section might be detrimental to their fragile health. The maternal factors are summarized in Table 2.

**Table 2:** Maternal characteristics of mothers of the cases and controls

**Variable**	**Case**	**%**	**Control**	**%**
CD4 ≤ 200 cells/μL	31	52	10	17
CD4 ≤ 350 cells/μL	44	70	30	50
Median CD4 count	180	IQR 131-361	357	IQR 229-435
STI during current pregnancy	18	30	10	17
VDS*	9		7	
Genital Herpes	6		2	
Genital Warts	3		1	
Mode of delivery				
Vaginal delivery	52	87	50	83
Elective ^ǂ^c-section	2	3	4	7
Emergency c-section	6	10	6	10
Mean duration of labour	8 hours	sd = 7	9 hours	sd = 5
Median period from rapture of membranes to delivery	5 minutes	IQR 5-45	10 minutes	IQR 5-25

*VDS = Gonorrhoea, Chlamydia, Trichomonas ^ǂ^C = caesarean.

### Nevirapine adherence

According to the pharmacy records, there were no stock outs of nevirapine for both mother and infant at the hospital during the study period. One hundred and nine (91%) had swallowed their single dose of nevirapine at onset of or during labour. There was high adherence to single dose of nevirapine among the respondents. Of the 11 who did not swallow the dose, three mentioned that the labour pains started when they were away from home and two mentioned that they did not think they were in labour. Three reported they did not get their dose since health workers’ were on strike during the time they meant to get the tablet. One hundred and thirteen (94%) babies swallowed nevirapine within 72 hours of delivery. The reasons given for non adherence for the newborns mentioned by three mothers were that they had a home delivery whilst two reported that the sisters forgot to give the baby before discharge.

### Infantile factors

Forty eight (80%) of cases and 40 (68%) controls were born at 37 weeks and above. More controls 43 (72%) than cases 36 (57%) had a birth weight of 2.500 kg.

Most of the respondents had practiced breast feeding. The reason given by all the women was that it was a cheaper option. The high cost of living and food shortages were the reason they opted for breast feeding.

All the women mentioned that they were taught during antenatal classes that not breast feeding reduces the transmission of HIV from mother to child. They were also taught that exclusive breast feeding was also encouraged as it reduces irritation of stomach lining which is experienced when there is mixed feeding or introduction of commercial milk products. From an ever recall definition of breastfeeding, thirty nine (65%) of both cases and controls had reported having practiced exclusive breast feeding for 6 months. Both the cases and controls had introduced mixed feeding at about the same time.

More cases 35 (59%) than controls 17 (29%) had been hospitalized in the last year. The main reason for the admissions was pneumonia. The immunization status for both the cases 48 (80%) and controls 55 (91%) was high. Reasons given for failing to immunize the children were that the baby was sick at the time of immunization was due and this was mentioned by five of the respondents. Three reported that the baby was still below the normal weight and has been failing to thrive. The rest reported that the baby had adverse effects at the last immunization.

### Risk factors

Factors that were found to be associated with HIV infection at Chitungwiza Hospital included maternal CD4 count of less than 200 during pregnancy [OR = 6.6 (95% CI 2.6-17)], breast feeding [OR = 4.3 (95% CI 1.1-16)] and those children who had been hospitalized since birth [OR = 3.6 (95% CI 1.7-7.8)]. Breast feeding exclusively for less than 6 months was found to be protective [OR = 0.4 (95% CI 0.2-0.9)]. Adherence to nevirapine was also found to reduce the risk of HIV infection among children [OR = 0.2 (95% CI 0.04-0.9)]. The factors associated with HIV infection analysed in this study are summarized in Table 3.

**Table 3:** Risk factors associated with HIV infection among children born to mothers on the PMTCT program in Chitungwiza, Zimbabwe 2008

**Factor**	**Cases**		**Controls**		**OR**	**95% CI**
	**Yes**	**No**	**Yes**	**No**		
Maternal CD4 count ≤ 200	28	32	7	53	6.6	2.59-16.91
Mixed feeding	57	3	49	11	4.3	1.13-16.17
Hospitalization	35	25	17	43	3.6	1.67-7.76
Exclusive breast feeding for ≤6 months	34	26	45	15	0.4	0.20-0.95
Nevirapine adherence	51	9	58	2	0.2	0.04-0.95
Full immunization status	46	14	55	5	0.3	0.08-0.92
Treated for an STI	18	42	10	50	2.1	0.89-5.29
Birth weight less than 2500 g	20	40	17	43	1.3	0.58-2.75
Caesarean section delivery	8	52	10	50	0.8	0.27-2.15
Pre-maturity	12	28	19	40	0.5	0.23-1.21
No breastfeeding	3	57	23	37	0.1	0.02-0.30

On performing stratified analysis, we found that the association between being hospitalized and risk of HIV infection was confounded by immunization. Those children who were hospitalized and who were not immunized were more likely to be HIV infected {OR = 11; 95% CI 0.6-187)}.

### Multivariate analysis

Further multivariate analysis (logistic regression analysis) was done to estimate the measures of association while simultaneously controlling for the confounding variables immunization. All the variables that were significant at the 0.25 level in the bivariate analysis were included in the logistic regression model using the stepwise method. We included the following variables in the stepwise model: Maternal CD4 count, no breastfeeding, treated for an STI, full immunization status, nevirapine adherence, and exclusive breast feeding for ≤6 months, hospitalization, and mixed feeding. Four independent factors appeared in the final logistic regression model. Breast feeding for ≤ six months independently reduced the risk of an HIV infection among children while having a CD4 count of ≤ 200 cells/μL during pregnancy, breast feeding and having been hospitalized, remained independent risk factors to HIV infection among children. Factors independently associated with HIV infection among children are shown in Table 4.

**Table 4:** Independent risk factors for contracting HIV infection among children born to mothers on PMTCT programme, 2008

**Variable**	**Cases**	**Controls**	**Crude OR**	**Adjusted OR**	**95%CI**	**p-value**
CD4 ≤ 200 during pregnancy	28	7	6.6	7.1	2.49– 20.36	<0.001
CD4 ≤ 200 during pregnancy	32	53			Reference	
Being hospitalized in childhood	35	17	3.6	2.9	1.17 – 4.81	0.020
Not hospitalized in childhood	25	43			Reference	
Exclusive breast feeding for ≤ 6 months	34	45	0.4	0.1	0.03– 0.41	0.001
	26	15			Reference	
Mixed feeding	57	49	4.3	29	4.19-207.85	<0.001
Not mix feeding	3	11			Reference	

## Discussion

In this study there are two major factors driving HIV infection in infants and these are breastfeeding and ARV prophylaxis. Factors affecting mother to child transmission of HIV can be divided into 5 categories (Table 5): (1) maternal factors (e.g. maternal immunologic status, anti-retroviral treatment), (2) virologic factors (e.g. viral load), (3) obstetric factors (e.g. traumatic delivery, chorioamnionitis, C-section), (4) fetal factors (e.g. prematurity) and (5) infant factors (e.g. immune status, breastfeeding, nutrition) [25].

**Table 5:** Description of various factors that influence mother to child transmission of HIV

**Factor**	**Description**
Viral	Viral genotype and phenotype, viral resistance and viral load
Maternal	Maternal immunological status, maternal nutritional status, maternal clinical status, behavioural factors and antiretroviral treatment
Obstetrical	Prolonged rupture of membranes (>4 hrs), mode of delivery, intrapartum haemorrhage, obstetrical procedures and invasive foetal monitoring
Foetal	Prematurity, genetic and multiple pregnancy
Infant	Breast feeding, gastrointestinal tract factors, immature immune system and ARV prophylaxis

This study found out that children born to mothers with a low CD4 count were at risk of HIV infection. A low CD4 count is an indicator of high viral load. Others studies had similar findings reporting women with CD4 counts less than 200 cells/μl were five times more likely to transmit HIV during breastfeeding [15,26,27]. Clinical trials have shown a strong positive correlation between maternal HIV viral load during pregnancy or at delivery and the risk of perinatal HIV transmission, even among women treated with ARVs [8]. In a study by Garcia P.M. et al. they concluded that in pregnant women with HIV-1 infection, the level of plasma HIV-1 RNA (viral load) predicts the risk but not the timing of transmission of HIV-1 to their infants [28,29]. Maternal viral load may be influenced by the mother’s immune responses resulting in different infection rates in infants and the infants may mount immune responses that protects them from infection [30].

HIV infection was higher in children who were breast fed. Avoiding breastfeeding would have been the best intervention if it was feasible. The duration of breast feeding has also an influence on HIV transmission. Early cessation of breastfeeding would decrease the child’s exposure to HIV through breast milk [18]. Those who were breast fed for less than six months were more protected than those who were breast fed for longer period of time. This finding could be site/setting specific as early cessation of breastfeeding by HIV infected mothers in Zambia did not improve the HIV-free survival among their infants [31]. Breast milk viral load increases following early abrupt cessation of breastfeeding. This poses greater risk of infection if infant returns to breast milk after abrupt cessation. Mothers need to be informed that once a decision of early abrupt cessation of breastfeeding is made, then the infant should not be re-introduced to breast milk again [32].

Studies have demonstrated that duration of breastfeeding is a major factor that drives postnatal HIV transmission. Most of the mothers (>65%) in this study breastfed their children beyond 6 months, which would be associated with higher postnatal HIV transmission to their infants [33]. In situations where breastfeeding cannot be avoided, extended ARV prophylaxis would help reduce risk of postnatal infection [34].

Most women had their children tested for HIV between six weeks to three months. This could have made the women with HIV positive women to continue breast feeding since their children would have contracted the HIV infection. The risk of MTCT through breastfeeding is cumulative. The longer the HIV-infected mother breastfeeds, the greater the additional risk of transmission through breastfeeding. This was supported in an observational study in South Africa, where they found out that exclusive breastfeeding during the first three months of life was associated with a lower transmission risk than mixed feeding [18,35].

Having been hospitalized during infancy was found to be independently associated with HIV infection among children born to HIV positive infected women. The most common cause of the hospitalisation was pneumonia, ARI and diarrhoea. Similar findings were observed in a study of 1266 children aged six months to five years hospitalized in Zambia where common causes of admission were pneumonia (28.4%), malaria (23.7%), malnutrition (18.3%), diarrhoea (10.4%), and tuberculosis (4.8%). Twenty-eight percent were seropositive for HIV-1 as opposed to 9.4% of children admitted with traumatic injuries. HIV-1 was found in 68.9% of the children with tuberculosis, 40.5% of those with malnutrition, 27.6% with pneumonia, and 23.7% of those with diarrhoea [36].

There were no differences in choice of delivery method by the mother since both cases and controls chose normal vaginal delivery. The study also found out that caesarean section reduces MTCT of HIV though this did not achieve statistical significance most likely due to the small number of mothers who delivered by this mode. Among both cases and controls delivery by caesarean section was not a popular method of delivery due to the increased hospital costs, longer hospital stay and fear that an operation would accelerate deterioration of their health. Caesarean section may not be promoted effectively in low resource settings like Zimbabwe as a public health intervention due to the costs and fears but can be offered as an option for those who can afford. Obstetric factors are known to affect the risk of transmission. A substantial number of cases of mother-to-child (vertical) transmission of human immunodeficiency virus type 1 (HIV-1) occur during the intrapartum period [37]. Possible mechanisms include transfusion of the mother’s blood to the foetus during labour contractions, infection after the rupture of membranes, and direct contact of the foetus with infected secretions or blood from the maternal genital tract [38]. Therefore, performing caesarean section before the onset of labor and the rupture of membranes could decrease the risk of vertical transmission. Early findings from on-going European cohort studies suggested an association between the mode of delivery and vertical transmission of HIV-1 [38-40].

Evidence from systematic reviews and other studies on the use of antiretroviral drugs to reduce the risk of mother to child transmission has shown that it is feasible achieve a clinically useful decrease in HIV transmission risk by lowering plasma viral load in pregnant women or when given as post-exposure prophylaxis in the newborns [41-44]. The effectiveness is pronounced in the early months and the focus is how to maintain this early treatment effect which is reduced when mothers continue to breast feed. Cessation of breastfeeding is impractical at an early stage for social, financial and hygienic reasons in low income settings [45,46]. It has been suggested to shorten the period of breastfeeding while continuing to administer preventive antiretroviral drugs to both the mother and uninfected infant in order to attain the maximum benefit [17].

### Limitations

The case control design is limited in that it can only show that there is an association but cannot determine causation. We therefore cannot attribute causation of HIV infection in children to any one of the factors studied. The study was carried out when most of the women had tested their children for HIV at six weeks. The data we obtained is subject to recall bias as women had to recall events from at least 18 months ago. We depended on reported data on breastfeeding (ever recall) which has potential pitfalls as mothers could have reported what they knew was the expected. We did not actively collect breast feeding data for the period we were studying. We noted a problem in the way the questionnaire was constructed, that it did not allow us to recode breastfeeding data into categories that were amenable to the assumptions of the regression model.

## Conclusions

Maternal CD4 count of ≤ 200 cells/μL, mixed feeding and being hospitalised in childhood were independently associated with HIV infection among children born to HIV positive mothers while exclusive breastfeeding for the first six months of live was protective. In the context of low resource settings, ANC counsellors must encourage exclusive breast feeding period for 6 months and stop breast feeding if acceptable, feasible, affordable, sustainable and safe. Early cessation of breastfeeding is associated with increased morbidity and mortality among infants even regardless of HIV exposure. HIV positive breastfeeding mothers and their infants should be give antiretroviral drugs to reduce the chances of infection through breast milk. The Ministry of Health and Child Welfare may need to change payment policy where HIV positive pregnant women opt to deliver via a caesarean section to prevent mother to child transmission and offer that service for free or at significantly subsidized fee.

## Competing interests

The authors declare no competing interests.

## Authors’ contributions

SN conceived of the study concept and design, developed the research protocol, acquisition, analysis and interpretation of data and wrote the draft manuscript. NTG: provided academic guidance with regards to conception, design, data collection, analysis, interpretation and reviewed several drafts and the final manuscript for important intellectual content. SM: conception, design, acquisition, analysis and interpretation of data and drafting the manuscript. MT provided overall technical and academic guidance and reviewed the final manuscript for important intellectual content. GS, AC conception, design, reviewing several drafts of the manuscript including the final draft for important intellectual content. All authors read and approved the final manuscript.

## Pre-publication history

The pre-publication history for this paper can be accessed here:

http://www.biomedcentral.com/1471-2458/13/1181/prepub
